# The Guarantee Mechanism of China’s Environmental Protection Strategy from the Perspective of Global Environmental Governance—Focusing on the Punishment of Environmental Pollution Crime in China

**DOI:** 10.3390/ijerph192214745

**Published:** 2022-11-09

**Authors:** Ran An, Tian Sang

**Affiliations:** 1 Law School, Qufu Normal University, Rizhao 276826, China; 2 Koguan Law School, Shanghai Jiaotong University, Shanghai 200030, China

**Keywords:** global environmental governance, China, ecological civilization, environmental crimes, environmental pollution crime

## Abstract

Effective global environmental governance is the only viable way to solve the human environmental crisis. For a long time, China has been an active promoter and contributor to the global environmental governance system. In recent years, China has enhanced the penalty intensity of environmental crimes, the environmental pollution crimes in particular, and received good results in order to better realize the construction of ecological civilization and better fulfill the emission reduction targets of international environmental treaties. The deterioration of China’s environmental crisis in the past and the lack of deterrent effect of China’s environmental laws are closely related to the ineffective punishment of environmental crimes. In order to better promote environmental protection careers, China’s environmental crimes still need to be continuously optimized in terms of adding charges, legislative models and restorative justice.

## 1. Introduction

The year 2022 marks the 50th anniversary of the first United Nations Conference on the Human Environment. Since then, the understanding of the environmental crisis in human society has been deepening. The environmental crisis is the alarm bell sounded by nature to all mankind. Global environmental governance is a key to solving the human environmental crisis [1]. At present, the environmental movements related to the world’s environment protection is still developing vigorously, and human awareness of environmental protection has increased significantly compared to the past. However, climate change, biodiversity loss, marine pollution, transboundary movement of hazardous waste and other international environmental crises are still serious, and the deep-rooted environmental conflicts between the international communities are becoming increasingly evident, thus global environmental governing bodies urgently need to build consensus and work together [2].

Global environmental governance has experienced ups and downs in recent years. In 2018, Japan announced that it would withdraw from the International Whaling Commission (IWC) and would restart commercial whaling in July 2019 [3]. As countries and regions around the world pay increasingly more attention to protecting the marine environment and biodiversity, the Japanese government’s sudden announcement of such a policy change shocked the world. In 2019, the United States suddenly withdrew from the Paris Agreement, which became one of the landmark events in the tortuous development of global environmental governance [4]. Although the United States rejoined the Paris Agreement after President Biden took office, the confidence of countries around the world to be able to work together to solve the environmental crisis may not be restored immediately [5]. In July 2022, due to the energy crisis that the Russian-Ukrainian conflict may bring to the European continent, Germany withdrew the “2035 energy industry greenhouse gas emission neutralization” target in the draft “Renewable Energy Law”, which is another iconic event highlighting the development dilemma of global environmental governance [6].

China is pushing forward the construction of ecological civilization with unprecedented intensity, and also contributing proposals to promote effective global environmental governance. In 2020, at the 75th session of the United Nations General Assembly, China announced its ambitious goal of peaking carbon emissions by 2030 and achieving carbon neutrality by 2060 [7]. In 2021, at the 15th Conference of the Parties to the Convention on Biological Diversity (CBD) in Kunming, Yunnan Province, China, Chinese leaders proposed a good vision of building “Three Earth Homes”. (At the conference, Chinese President Xi Jinping proposed the vision of building an earth home where people and nature live in harmony; an earth home where the economy and the environment work together; and an earth home where all countries in the world develop together. See the website of the Central People’s Government of China [8]). What measures does China take to ensure the realization of these ambitious goals? As a matter of fact, China’s environmental protection progress in the past 10 years is closely related to the effective punishment of environmental crimes in China, which has become the most powerful guarantee for China’s environmental protection cause. China’s environmental crime law mentioned in this article refers to Chapter VI of the specific provisions of criminal law of China (Articles 338–346). It is no exaggeration to say that China’s recent changes in the punishment of environmental crimes are a concentrated expression of China’s determination to protect the environment. Through the investigation of China’s punishment of environmental crimes, we can learn about the guarantee mechanism of China’s environmental protection cause, about the development trend of China’s environmental protection, and learn how China has effectively set an example for global environmental governance with its own actions.

Environmental law is the main mechanism for carrying out environmental protection strategy and policy, and environmental crime is the most serious act among many environmental pollution behaviors, which most requires legal punishment. Since the 1950s, several countries have begun to attach importance to the application of criminal law to combat environmental pollution. Leading environmental protection countries such as Germany, the Netherlands, Finland, and Spain have all enacted environmental crime legislation [9]. Because the pollution result of environmental crime has international mobility, countries around the world pay more and more attention and research on environmental crime. China promulgated the environmental criminal law in 1997. Therefore, countries with successful experience in punishing environmental crimes have become the main research objects of China. In particular, since Germany and China are both civil law countries, German environmental criminal legislation and its judicial practice have become an important reference for China over the past decades. This article will mainly use comparative research methods to discuss the formulation process of German environmental criminal law, as well as the application of these experiences to China.

Since the reform and opening up in the late 1970s, China’s economic development has made great achievements, but it has paid a serious environmental price which has attracted worldwide attention [10,11,12]. Especially after joining the World Trade Organization (WTO), China’s environmental issues have received more global attention, and an increasing number of articles have been written about the development of China’s environmental law, but few academic articles have systematically introduced China’s environmental crimes and those previous academic articles also need to be updated [13,14,15]. Therefore, the aim of this paper is to systematically explain the process of the promulgation and revision of China’s environmental criminal law as well as its judicial practice. This paper first discusses the development of global environmental governance and the evolution of China’s environmental protection strategy. This is the external background of China’s current significant environmental pressure and the internal impetus for building ecological civilization. It is also an indispensable historical background for studying the necessity of environmental crime punishment in China. In the second part, this paper expounds on the development process and judicial practice of China’s environmental criminal law. We can see that in the past decade, it is precisely because of the increased punishment of environmental crimes that China’s environmental governance strategies and programs have been strongly supported, and China’s environmental protection has thus made many important achievements. Next, because the theme of this paper has an obvious theoretical inclination, it could not use quantitative analysis. Therefore, the comparative research method is the main research method of this paper. Therefore, this paper discusses the legislative and judicial experience of environmental crime in Germany in detail, and briefly introduces the experience of environmental crime punishment in Japan, as well as the theoretical analysis of relevant foreign experience by Chinese scholars. Finally, this paper proposes that the punishment of environmental crimes in China should learn from the encouraging experience of environmental pioneer countries such as Germany and Japan, and puts forward more detailed improvement directions and suggestions for the future improvement of the punishment of environmental crimes in China.

## 2. The Development of Global Environmental Governance and the Evolution of China’s Environmental Protection Strategy

The global spread of the environmental crisis has made mankind fully aware of the need for and urgency to work together with regard to environmental protection. Global environmental governance has come a long way since the environmental movement began in the 1960s and 1970s, and has taken on different characteristics at different times in history. At the same time, China’s environmental responsibilities are constantly evolving, and the pressure on China to protect the environment have also increased internationally. How to ensure that China’s environmental protection strategies and policies can be truly implemented has become a more prominent issue.

### 2.1. Development Stages and Characteristics of Global Environmental Governance

Since human beings entered the period of industrial revolution, especially the second one, environmental crises have continuously emerged. Environmental pollution incidents such as the smog in Los Angeles, the smog in London, and the Minamata disease in Japan shocked the whole world. The inherent unsteadiness of environmental crises has put all countries at risk. In 1972, the convening of the first United Nations Conference on the Human Environment was a milestone in global environmental governance. Taking this as a starting point, global environmental governance can be roughly divided into three phases with different characteristics.

#### 2.1.1. Phase 1: Led by Developed Countries, Presenting a North-South Division Pattern (1972–1992)

After the Stockholm Conference on the Human Environment in 1972, substantial progress had been made in global environmental governance. At this stage, developed countries in Europe and the United States have actively promoted international environmental cooperation due to their advantages in environmental protection concepts, environmental protection technology and financial support, and objectively played a leading role in global environmental governance. A series of the most important international treaties in the cause of human environmental protection were signed at this stage, such as the *Declaration of the United Nations Conference on the Human Environment* (1972), the *Convention on International Trade in Endangered Species of Wild Fauna and Flora* (1973), the *Vienna Convention for the Protection of the Ozone Layer* (1985), the *Convention on Assistance in the Case of a Nuclear Accident or Radiological Emergency* (1986), and the *Basel Convention on the Control of Transboundary Movements of Hazardous Wastes and Their Disposal*(1989), etc. At this stage, two camps of global environmental governance have gradually formed, namely the camp of developed countries and the camp of developing countries, referred to as the “North and South”. There is a significant gap between the North and the South in terms of economic strength and ability to respond to environmental crises, and the environmental protection demands of the two are also quite different. Developed countries in Europe and the United States are more willing to take strong measures, while developing countries express that their own environmental problems are largely caused by insufficient development, and they argue that they cannot undertake environmental protection measures that do not match their national strength [16]. At this stage, developed countries began to introduce environmental crime regulations one after another to ensure the implementation of environmental laws with such powerful measures.

#### 2.1.2. Phase 2: The Swaying Attitude of Developed Countries, the Formation of a Three-Legged Separation Pattern (1992–2015)

In the context of the continuous deterioration of the global environment and the more serious development-related issues, the United Nations Conference on Environment and Development was held in Rio de Janeiro, Brazil in 1992. This conference is another milestone in the course of human environmental protection. At the meeting, the *Rio Declaration on Environment and Development*, *Agenda 21*, and the *Statement of Principles on Forests* were adopted, the *United Nations Framework Convention on Climate Change* and the *United Nations Convention on Biological Diversity* were opened for signature, the international environmental protection principle of “common but differentiated responsibilities” was formally proposed and officially confirmed in Article 10 of the *Kyoto Protocol* (1997). However, the global environmental governance at this stage was not all smooth sailing. In 2001, US President Bush announced the US’s intention to withdraw from the *Kyoto Protocol*. Subsequently, Japan, New Zealand, Canada and some other countries also indicated that they would no longer participate in the Kyoto agenda. At this stage, the EU has made many efforts to promote global environmental governance and played an active leading role. In a nutshell, global environmental governance at this stage is roughly reflected in the three-legged separation of the “Umbrella Group” dominated by the EU and the United States and the “G77 and China” [17]. At this stage, environmental criminal laws in developing countries have gradually emerged or improved, keeping up with the environmental trends of developed countries.

#### 2.1.3. Phase 3: The Gradual Movement of Developing Countries to the Center has Emerged (2015–Present)

In 2015, the signing of the *Paris Agreement* opened a new model of global environmental governance: the bottom-up model. Developing countries are increasingly responsible for international environmental protection, while the leadership of Europe and the United States in international environmental cooperation appears to have some stamina. The sudden withdrawal of the United States from the *Paris Agreement* in 2017, has seriously dampened the confidence of global environmental governance, and created a leadership gap in international environmental cooperation [18]. Under such circumstances, the Chinese government has made it clear that it will firmly safeguard global environmental governance and continue to promote the signing and implementation of follow-up agreements to the *Paris Agreement*. The United States announced its return to the *Paris Agreement* after President Biden took office, bringing a new boost to the troubled global environmental governance. However, as the world’s superpower, the changing attitudes with regard to the international environmental protection issues of the United States have made countries around the world question the original international environmental consultation mechanism, and they have also begun to think about the framework and mechanism of a new type of global environmental governance [19]. Under this realistic background, it becomes more and more important for developing countries to better solve their own environmental problems and express their determination to protect the environment. Hence, environmental crime legislation in developing countries will take on greater responsibility in ensuring the implementation of environmental laws.

### 2.2. The International Environmental Situation Facing China

In 1971, China resumed its legitimate seat at the United Nations, it has never been absent from major international environmental conferences, and China is also a member of many important international environmental treaties. Over the years, China has been committed to promoting the global environmental governance system towards a more fair and reasonable direction of win-win cooperation. At present, the effect and future of China’s environmental protection work is still facing great international pressure, the “China environmental threat theory” still appears frequently in the field of international public opinion, and the green barriers in international trade have perplexed China for a long time.

First of all, China is a well-deserved manufacturing power, and the added value of manufacturing has ranked first in the world for 12 consecutive years [20]. Although such a large-scale manufacturing productivity is an important driving force for China’s economic development, it also brings huge environmental pressure. In 2019, China’s annual greenhouse gas emissions accounted for 27% of the world’s total, surpassing the total emissions of the *Organization for Economic Co-operation and Development* (OECD) countries for the first time. Although China’s per capita emissions are still lower than those of developed countries, (for example, China’s per capita emissions of 7.1 tons in 2019 are still significantly lower than the U.S. per capita emissions of 16.1 tons, See Zhang [21].) it is an indisputable fact that China still faces enormous environmental pressure from the world. In the mid-1990s, the “China Environmental Threat Theory” appeared in the international media and academia. Some Western researchers believe that China’s rapid economic development comes at the cost of massive consumption of natural resources and a heavy blow to the ecological environment. If China and India reach the developed countries’ standard of living, the world’s natural resources will only be sufficient for these two countries [22]. In 2007, the *New York Times* did a series of 10 reports on China’s environmental crisis with the title of *Choking on Growth: China’s Environmental Crisis*, simplifying the complex environmental crisis and even blaming China for the rise in international oil prices. Today, unfriendly voices that turn a blind eye to the development of China’s environmental protection cause still frequently appear in international public opinion [23].

Secondly, China’s foreign political and economic cooperation also needs to focus more on environmental cooperation. China has seen rapid economic development since its accession to the WTO in 2001, and has been one of the biggest beneficiaries of globalization in the last four decades. In recent years, the wave of globalization has suffered some setbacks and a resurgence of international conservatism, but China remains an important supporter of globalization. Environmental issues have become increasingly important in China’s foreign cooperation, as the practice of transferring backward production capacity from developed countries to developing countries is losing popularity, and a new model of international environmental cooperation needs to be built. For example, environmental protection has been given high priority in China’s “Belt and Road Initiative” international cooperation strategy. In 2017, the Chinese government issued the *“Belt and Road Initiative” Ecological and Environmental Protection Cooperation Plan*. In March 2022, China also issued the *Guidance on Promoting Green “Belt and Road Initiative”*, which proposed detailed tasks on cooperation in key areas of green development and green development of overseas projects during the implementation of the “Belt and Road Initiative”. In short, under the complicated international environmental protection situation, there is no turning back for China’s environmental protection work, and it must stride forward along the road of green development.

### 2.3. The Focus of China’s Environmental Protection Strategy in the New Era

Needless to say, China’s environmental issues were once very serious. In the past decade or so, especially since the new generation of Chinese government came to power, China has been rectifying environmental problems with unprecedented determination and intensity, and has received good results. However, the overall environmental situation in China is still unsatisfactory. The *China Environment Status Bulletin 2019* shows that 180 of China’s 337 cities at the prefecture level and above, or 53.4% of the total, have ambient air quality that exceeds the standard. A total of 452 days of heavy pollution occurred in 337 cities, 183 days less than in 2018; there were also 1666 days of severe pollution, 88 days more than in 2018. The national Ecological Environment Index (EI) value was 51.3, and the area of counties with general, bad and worse raw ecological quality accounted for 55.3% of the national territory. (The 2019 *China Environment Status Bulletin* can be downloaded from the website of the Ministry of Ecology and Environment of China [24].) It can be seen that the challenges facing the construction of ecological civilization in China are still severe.

With the rapid development of China’s economy, the income of Chinese people continues to rise. China’s per capita GDP reached 12500 US dollars in 2021, which has reached the upper limit of upper-middle-income countries and is about to enter the ranks of high-income countries [25]. As we all know, the rapid development of the economy will lead to changes in values. The value orientation of Chinese society has gradually shifted from material needs such as the possession of economic wealth to richer spiritual needs such as longing for quality of life and self-realization. A good ecological environment is the basis of a better life, and the Chinese people pay more and more attention to environmental protection [26]. Therefore, China’s domestic political tasks and economic development goals have also changed accordingly, and creating a good ecological environment is not only the realistic requirement of complying with the objective laws of nature, but also an important basis for realizing the harmonious development of Chinese society. It has become the consensus of the Chinese authorities. In this context, the cause of environmental protection is getting unprecedented attention in China.

Since the 18th Plenary Congress of the Communist Party of China, China has entered a new era of socialism with Chinese characteristics. One of the biggest highlights of China’s new era is to give ecological civilization construction an equally important strategic position as economic construction. In 2018, the concept of ecological civilization was officially included in China’s *Constitution*, and China’s ecological civilization construction entered a new pattern of “environmental constitution” [27]. In the latest Chinese *Constitution*, the characteristics of China’s development are described as “promoting the coordinated development of material civilization, political civilization, spiritual civilization, social civilization, and ecological civilization”, while the goal of national development has been optimized from “building our country into a prosperous, strong, democratic and civilized socialist country” in the early 21st century to “building our country into a prosperous, strong, democratic, civilized, harmonious and beautiful modern socialist country”. Among these goals, the main connotation of “beauty” is to have a beautiful ecological and natural environment [28]. It can be seen that China’s determination towards the construction of ecological civilization is unprecedented, and the concept of ecological civilization is also being thoroughly implemented in all fields of China’s rule of law. For example, the epoch-making *Civil Code* of China, which came into force in 2021, stipulates the green principles, (Article 9 of China’s *Civil Code* stipulates that “civil subjects engaged in civil activities shall be conducive to saving resources and protecting the ecological environment”.) making the concept of green development a basic principle guiding China’s civil activities. Another example is that China has officially started the compilation of the “*Environmental Code*” in 2021, which will greatly enhance the independent status of environmental law in China’s rule of law, and is another excellent example of China’s determination to protect the environment [29].

## 3. Development Context and Practical Effects of China’s Legislation on Environmental Pollution Crime

Compared with western developed countries, China’s environmental crime legislation came into force relatively late. In 1997, when China overhauled its *Criminal Law*, it added provisions for environmental crimes. However, during the period from 1997 to 2011, China’s punishment for environmental crimes was very limited, which objectively resulted in the lack of intensity of China’s environmental law as a whole, and did not form a sufficient deterrent to those polluters. Environmental Pollution Crime is the core charge of China’s environmental crime legislation. This article will mainly take the legislative evolution and judicial practice effect of this crime as an example to explain how China’s punishment of environmental crimes ensures the implementation of China’s environmental protection strategy.

### 3.1. From Major Environmental Pollution Accident Crime to Environmental Pollution Crime

In 1997, China undertook a major revision of the *Criminal Law*. In Chapter 6 of the *Criminal Law*, “Crimes against the Order of Social Administration”, the legislator created a special section for crimes against the protection of resources and the environment, with a total of 9 articles and 14 crimes, which officially started the history of environmental crime legislation in China. The most notable provision is the first article in this section, namely the crime of “Major Environmental Pollution Accident Crime” (Before 2011, the “crime of major environmental pollution accident” stipulated in Article 338 of China’s *Criminal Law*, its content was “in violation of state regulations, the discharge, dumping or disposal of radioactive wastes, containing infectious disease pathogens into land, water bodies, and atmosphere. If a serious environmental pollution accident is caused, causing heavy losses to public or private property or serious consequences of personal injury or death, the sentence shall be fixed-term imprisonment of not more than three years or criminal detention, and a fine or a fine; if the consequences are particularly serious, shall be sentenced to fixed-term imprisonment of not less than three years but not more than seven years, and shall also be fined.”) under Article 338 of the *Criminal Law*. The crime of “Major Environmental Pollution Accident” is the predecessor of the crime of “Environmental Pollution” that is now receiving attention in China. Due to the fact that Chinese society did not have a clear understanding of the problem of environmental pollution, and did not clearly recognize the great harm that environmental pollution could bring, or perhaps due to the realistic needs of the rapid economic and social development at that time, the legislator limited the subjective form of the crime of major environmental pollution accident to negligence. In other words, according to the principle of criminality, the act of intentionally polluting the environment could not constitute this crime, which led to the delay in the full application of the crime of major environmental pollution accidents for many years. There are less than 50 cases of this crime in more than ten years after its introduction, and the social media have been reporting and analyzing this problem in depth, and academia has criticized it. (See Jiao [30]. According to the statistics in the article, the number of environmental crime cases in China between 2000 and 2010 was only 39. Therefore, at this stage, it is impossible for China’s environmental crime legislation to have the deterrent effect it should have.)

With the growing awareness of environmental protection among the Chinese public, Chinese society is becoming less tolerant of environmental pollution. Against this backdrop, China enacted the eighth amendment to its *Criminal Law* in 2011, the *Criminal Law Amendment (VIII)*. One of the major highlights of the *Amendment (VIII)* was the significant amendment to Article 338 of the *Criminal Law*, which changed the term “Major Environmental Pollution Accident Crime” to “Environmental Pollution Crime”. The change in the name of the crime shows the determination of Chinese lawmakers to use criminal means to protect the environment. (In 2011, after the promulgation of China’s *Criminal Law Amendment (VIII)*, the “crime of major environmental pollution accident” was adjusted to “crime of environmental pollution”, the content of which is “violating state regulations, discharging, dumping or disposing of radioactive waste, Whoever contains wastes, toxic substances or other harmful substances containing pathogens of infectious diseases and seriously pollutes the environment shall be sentenced to fixed-term imprisonment of not more than three years or criminal detention, and shall also or only be fined; if the consequences are especially serious, he shall be sentenced to fixed-term imprisonment of not less than three years but not more than seven years, and fined.”) After that, *the Criminal Law Amendment (VIII)*, which came into effect in March 2021, increased the statutory penalty for the four cases of particularly serious pollution to more than seven years of imprisonment, further demonstrating China’s determination to build an ecological civilization.

From the wording of the crime of environmental pollution it can be clearly seen that pollution of the environment is no longer considered an “accident”, but rather an intentional action. This has significantly lowered the threshold for criminal law to regulate serious environmental pollution, and all sectors of society have appreciated this change in the Criminal Law Amendment (VIII). However, due to the limited wording, the correct understanding of “seriously polluting the environment” became the biggest obstacle for prosecutors and courts to apply this crime. Two years after the introduction of this crime, there are still very few cases of its application by Chinese courts, and the gap between this crime and the expectation of the society is very obvious. Compared to the crime of major environmental pollution accidents, the crime of polluting the environment does not seem to play a more effective role. However, the situation improved significantly after 2013.

### 3.2. The Important Role of Judicial Interpretations on Environmental Pollution Crimes

In view of the delayed state of the judicial application of environmental pollution crimes to meet social expectations, the Supreme People’s Court of China and the Supreme People’s Procuratorate jointly issued the *Interpretation on Several Issues Concerning the Application of Law in the Handling of Criminal Cases of Environmental Pollution* (hereinafter referred to as the *Interpretation*) in June 2013. It is considered in *Interpretation* that the following circumstances should be identified to have “severely polluted the environment “: 1. Discharging, dumping, or disposing of radioactive wastes, wastes containing pathogens of infectious diseases, or toxic substances in the first-level protected areas of drinking water sources or the core areas of nature reserves; 2. Illegally discharging, dumping or disposing of more than three tons of hazardous waste; 3. Illegal discharging of pollutants containing heavy metals, persistent organic pollutants and other pollutants that seriously endanger the environment or harm human health exceed more than three times of the national pollutant discharge standards or the pollutant discharge standards formulated by the people’s governments of provinces, autonomous regions and municipalities directly under the Central Government in accordance with the authorization of the law, etc.,. (Other representative identification marks of serious environmental pollution include causing the interruption of a water intake from centralized drinking water sources above townships for more than 12 h; causing the loss of public and private property of more than 300,000 yuan; causing the evacuation or transfer of more than 5000 people; Causing more than 30 people to be poisoned; causing minor injury, mild disability, or organ tissue damage to more than three people leading to general dysfunction; causing serious injury, moderate disability, or organ tissue damage leading to serious dysfunction of more than one person.)

The promulgation of the *Interpretation* has completely broken the “seal” of Article 338 of the *Criminal Law* for nearly two decades and immediately set off a wave of cases of Environmental Pollution Crime throughout China. This is hundreds of times the number of cases before the promulgation of this *Interpretation*. (The data comes from the China Judgment Document Network (https://wenshu.court.gov.cn (accessed on 20 May 2022) established by the Supreme People’s Court of China. As of 20 May 2022, the website has included 12,448 criminal judgments for the crime of environmental pollution.) Although judicial interpretation is not a law in the strict sense, under China’s current rule of law system, it is difficult to deny that judicial interpretation has the nature and status of “quasi-legislation”. From the perspective of academic research, the promulgation of the *Interpretation* is actually a more detailed instruction of Standard of incrimination of Environmental Pollution Crime, while weakening the importance of the causal relationship in the judicial application of Environmental Pollution Crime, thus making the application of the crime more clear and flexible, although some scholars argue that this approach may be suspected of violating the principle of criminal law modesty by bringing the *Criminal Law* out prematurely [31]. However, in the context of China’s growing environmental crisis and the sudden increase in public demand for environmental protection, it is reasonable for legislators to take certain contingency measures in the embarrassing situation of having no case to convict for environmental pollution crimes.

### 3.3. The Punishment of Environmental Pollution Crime and the Implementation of China’s Environmental Protection Strategy

Through nearly 20 years of exploration, China has worked out a safeguard mechanism that adapts to China’s environmental protection strategy. The core method is to increase the punishment intensity of environmental crimes so as to increase the deterrence of China’s environmental law system and cultivate public awareness of environmental protection.

China’s previous one-sided development concept of economic growth is the root cause of China’s environmental crisis failure. In fact, China’s introduction of environmental legislation was not too late, and in 1979 China promulgated the *Environmental Protection Law (for Trial Implementation)*. Since then, the pace of enacting environmental laws in China has been accelerating, with dozens of slip environmental protection laws and administrative regulations coming out one after another, with the State Council and local governments promulgating hundreds of environmental protection administrative rules [32]. However, China’s punishment intensity of environmental crimes has not been as strong as it should be, resulting in an overall lack of deterrence in China’s environmental law system. The economic construction-centered development philosophy has led environmental protection departments throughout China to mostly use administrative penalties in the form of fines for environmental pollution caused by enterprises whose profits are often far greater than the fines, resulting in enterprises preferring to pay fines rather than eliminate outdated production capacity and to purchase expensive environmental protection devices. In China, the public security authorities are responsible for filing and investigating environmental crimes, and the public security authorities in China are responsible for a wide range of matters, and the problem of many cases and few people is very prominent. In this situation, the public security organs, on the one hand, lack knowledge of environmental protection technology and do not work smoothly with the environmental protection departments; on the other hand, under the influence of the one-sided emphasis on the concept of economic development, the public security organs also lack the motivation to take the initiative to punish environmental crimes. This has lead Chinese environmental crime legislation to become “zombie provisions”. For example, prior to 2010, in the southern part of Jiangsu province, where China’s economy is most prosperous and industrial enterprises are most concentrated, the grassroots courts basically did not adjudicate environmental crime cases [33].

It can be seen from the above that it is difficult to effectively alleviate the environmental crisis in China when the punishment intensity of environmental crimes is weak. In the early 21st century, environmental pollution incidents such as river pollution in Songhua River, sewage leakage of Zijin Mining in Fujian, and sandstorms and smog have greatly warned the Chinese people, and also greatly enhanced the environmental protection awareness of the Chinese government and society. The previous one-sided pursuit of economic growth and material wealth has been deeply reflected, and the status of ecological civilization construction in China’s national development strategy has been continuously improved. After the advent of the Environmental Pollution Crime in 2011, especially after the release of the *Interpretation* in 2013, the large-scale implementation of Environmental Pollution Crime in judicial practice greatly deterred various entities that cause environmental pollution, and it effectively curbed China’s environmental crisis continues to worsen. Taking China’s Beijing-Tianjin-Hebei region as an example, in 2019, the number of good weather days in this region and surrounding “2 + 26” cities has increased to 53.1%. Especially in Beijing, the proportion of days with air quality reaching the standard in 2019 was 65.8%, and there are no heavily polluted days for the first time in the whole year [34]. Therefore, the effective implementation of China’s environmental protection strategy must be guaranteed by the effective punishment of environmental crimes.

## 4. Insufficiency and Optimization Direction of Punishment of Environmental Crimes in China

Although the legislation and judicial practice of environmental crimes in China are basically on the right track, there is still a lot of room for optimization. After entering the 21st century, the German criminal law and its theoretical research have had an increasingly profound influence on the formulation and theoretical research of Chinese criminal law. In recent years, Chinese scholars have conducted a comprehensive and in-depth study of German environmental criminal law, and regarded it as an important reference for punishing environmental crimes in China [35,36,37,38].

### 4.1. The Development and Reference Value of German Environmental Criminal Law

German criminal legislation has a long history of focusing on environmental interests. As early as 1871, the Prussian Penal Code included provisions for the crime of maltreating animals [39]. Of course, environmental criminal legislation at this historical stage is still very fragmented and does not reflect modern environmental protection concepts. Like most environmentally friendly developed countries, Germany really realized that it was only after World War II that it was necessary to use criminal law to deal with the environmental crisis. At that time, Germany’s industrial development not only quickly reached the level before the war, but also made great progress, especially in terms of heavy industry such as automobile, steel, and machinery manufacturing, for which it became the global industry leader. However, the rapid development of industry has also brought serious environmental pollution to Germany [40,41,42].

Under this context, Germany has promulgated hundreds of laws and regulations related to environmental protection in a short period of time, but they have not achieved good environmental protection effects, and have instead led to confusion in the application of laws and the responsibilities of environmental protection agencies. German legislators decided to change this chaotic status quo. According to the draft amendments to the criminal law put forward by various experts in 1971, the German legislature passed the 18th Criminal Code Amendment Act on 28 March, 1980, which added to the German Criminal Code [43]. The special chapter of “Crimes against the Environment”, that is, Chapter 28 of the Criminal Law, absorbs some criminal provisions in administrative environmental protection laws, and adds some new crimes, such as water pollution, air pollution, noise pollution, waste pollution, etc. [44]. The addition of a special chapter on environmental crimes to the criminal law has accomplished two legislative purposes. First, through this amendment, Germany has significantly expanded the scope of using criminal law to investigate acts of environmental damage and strengthened the deterrence of national laws against environmental pollution. Second, through the revision of the law, it reflects the progress of the national environmental protection concept, the development trend from the original anthropocentrism to the eco-centrism, and at the same time stimulates the national awareness of environmental protection and makes the German people more actively participate in the work of punishing environmental crimes.

The special chapter on environmental crime added by Germany in 1980 promoted the unification and standardization of environmental crime punishment in Germany to a certain extent, but the law still did not solve the dependence of environmental crime legislation on administrative law, which led to certain criticisms [45]. Under the concerns of the government and the public, the revised “Anti-Environmental Crime Act” came into effect on 1 November 1994, specifically the thirty-first amendment to the German Criminal Code, which further expanded the scope of environmental crimes and enhanced punishment [46]. The revised German Penal Code in 1998 then shifted the position of environmental crimes to Chapter 29 of the Criminal Code. The specific crimes in this chapter include polluting waters (Article 324), polluting land (Article 324a), air pollution (Article 325), causing noise, vibration and Nonionic radiation (Article 325a), the unauthorized disposal of garbage (Article 326), the unauthorized operation of nuclear equipment (Article 327), the unauthorized trade in radioactive substances and other dangerous goods (Article 328), the infringement of protected areas (Article 329), crimes against the environment with particularly serious circumstances (Article 330), poisoning causing serious harm (Article 330a), etc. Thereafter, German environmental crime regulations have basically stabilized.

German environmental crime legislation has also encountered some doubts and criticisms. For example, the provisions of the German environmental criminal law are relatively vague and must be combined with the corresponding environmental administrative provisions with regard to conviction and sentencing. Therefore, this is equivalent to transferring the power of criminal legislation and justice to the ministry of environmental protection [47]. At the same time, because many environmental administrative regulations are complex and difficult to understand, this legislative model also makes the punishment of environmental crimes ambiguous, which violates the principle of clarity required by modern criminal law. There are also views that the effect of applying criminal law to combating environmental pollution is difficult to measure and may violate human rights in other fields. There is also a view that environmental crime legislation is a legislative trend spawned by the modern environmental movement, and the use of criminal law to protect the environment may actually be symbolic legislation which only reflects the state’s attitude towards environmental protection [48].

However, the anti-criticism points out that environmental problems are very different from traditional personal and property crimes, and the cumulative and fluid characteristics of environmental problems make them inseparable from the determination of science. Various environmental protection standards often exist in environmental protection administrative regulations, which is a common practice in all countries in the world, and it is impossible for the criminal law to stipulate these contents in detail [43]. Some ingenious research has confirmed that the German environmental criminal law does have practical effects, and the deterrent force of the criminal law obviously helps to strengthen citizens’ awareness of environmental protection [49].

Under the protection of German environmental criminal law, the German environmental law system (including relevant EU environmental laws) has made great contributions to the improvement of the German ecological environment. Germany once also faced a serious environmental crisis, especially the industrial enterprises along the Rhine River which directly discharged industrial wastewater into the river, resulting in serious river pollution [50,51]. Germany’s environmental situation has improved significantly since 1990, and Germany has achieved a lot in the field of climate action. Germany’s emission of greenhouse gases fell by 23.8% between 1990 and 2013, and eutrophying and acidifying air pollutants and of ozone precursors decreased to 60% of their 1990 level by 2012 [52]. In 2019, about 43 percent of electricity was generated from renewable sources, such as wind and solar power [53]. At present, Germany is one of the most sustainable industrial countries. Many companies are committing to their social responsibility [54]. Internationally, Germany leads the way in climate protection and is a pioneer in the development of renewable energies [55].

To sum up, Germany’s environmental protection achievements over the past half century are universally recognized. From an empirical perspective, a responsible conclusion can also be drawn, that is, without the safeguarding role of German environmental criminal law, it is difficult for the implementation of German environmental law to be so strong. In fact, this is also the greatest significance of the existence of criminal law, because although civil law and administrative law can deal with many social problems, if there is no strong guarantee of criminal law, it is difficult for these laws to be effectively followed. The development of German environmental criminal law has brought great enlightenment value to the improvement of China’s environmental criminal law, especially with regard to issues such as legislation, new crimes, and subjective form(mens rea).

In addition to Germany, Japan’s experience in environmental crime legislation is also of great reference value to China. Japan and China, both Asian countries, are also deeply influenced by Confucianism. After the Second World War, Japan achieved rapid economic development, and even ranked second in the world in terms of GDP for more than four decades. [56] This situation is very similar to China today. [57] In the middle and late 20th century, with the rapid development of Japan’s economy, its environmental crisis has reached a very dangerous level, and half of the world’s eight major public hazard crises occurred in Japan. [58] Under this situation, Japan convened the 64th Congress in 1970, and formally promulgated a series of environmental protection laws, including Japan’s first environmental criminal law. [59]

In terms of legislation, unlike Germany’s practice of incorporating environmental crime legislation into the criminal code, Japan’s environmental criminal law is a special criminal law, that is, special provisions of the criminal law are promulgated outside the Japanese criminal code. The main feature of Japanese environmental criminal law in content is that, first of all, it was earlier stipulated in Asian countries that both natural persons and enterprises can be the subject of environmental crimes. Secondly, Japan’s environmental criminal law also clearly distinguishes the subjective mentality of intentional and negligent environmental crimes, which makes the punishment scope of environmental criminal law very strict. Thirdly, the characteristic of Japanese environmental criminal law is that between the discharge of harmful substances and the specific dangerous state, there is no need to prove a specific causal relationship, and the presumption of causal relationship is set, which reduces the difficulty for the court to convict the defendant. [60] Through decades of environmental protection efforts, Japan has become one of the most beautiful and safe countries in Asia. The water quality of Japan’s main rivers is even higher than that of European and American countries. [61]

### 4.2. The Optimizing Direction of Environmental Crime Punishment in China

China, Germany and Japan are all civil law countries. Germany and Japan have all experienced the environmental protection process of “pollution first, protection later”. Therefore, the relevant experience of Germany and Japan provides a quite suitable reference for the punishment of environmental crimes in China. In order to achieve the ambitious goals of carbon peaking in 2030 and carbon neutrality in 2060, the punishment of environmental crimes in China should focus on the following aspects.

First, China’s environmental legislation still needs to add new charges at the legislative level. At present, China’s environmental criminal legislation has relatively mature control over water pollution, air pollution, solid waste pollution, and the destruction of wildlife resources. However, the punishment for environmental pollution or destruction such as noise pollution, destruction of grasslands, and destruction of wetlands is still far from sufficient. With the continuous advancement of urbanization in China, the problem of noise pollution has become more and more serious, threatening the physical and mental health of people living in cities, particularly large ones [62]. Natural elements such as grasslands and wetlands that have not been included in the scope of environmental crime protection are of great significance to ecological balance and soil and water conservation. Therefore, the legislative expansion of China’s environmental criminal legislation is very necessary.

Second, the subjective form (mens rea) of environmental crime legislation in China is in urgent need of improvement. Before 2011, the core crime of environmental crime in China was the crime of major environmental pollution accident, and the subjective form of this crime was negligence. After 2011, the crime was adjusted to environmental pollution crime, but there is no official description of the subjective form of the new crime, which has led to controversial adjudication in judicial practice, as both intention and negligence can constitute this crime. According to the tradition of Chinese criminal law, this is obviously unreasonable. In German environmental criminal law, the statutory penalty for intentional environmental crime and negligent environmental crime is clearly distinguished. This legislative method not only made the criminal law more airtight, but also clarified the law for judicial personnel and calmed academic disputes at the same time.

Third, the legislative model of environmental crime legislation in China should be optimized. At present, China’s environmental crime legislation is uniformly stipulated in the *Criminal Law*, and this model was not too problematic when China’s *Criminal Law* was revised in 1997. However, over time, the drawbacks of this model are gradually exposed. On one hand, China’s environmental crimes fall into a category of Chapter VI of the *Criminal Law* of China, “Crimes of Obstructing Social Management Order”, which lacks independence and cannot show the significance of environmental crime punishment to China’s environmental protection strategy. Moreover, the viewing of environmental crime as an act that hinders the order of social management is also a reflection of China’s previous one-sided emphasis on economic construction [63]. On the other hand, the existing legislative model makes the connection between China’s environmental crime and China’s environmental law system poorly. For example, how the environmental concept in criminal law and in environmental law are docked and integrated, how the ecological protection boundary of criminal law is delineated, and how the criminal law keeps up with the speed of updating environmental law, etc. These issues have always plagued legislators and theoretical circles [37]. Currently, some scholars suggest that China can imitate Japan and use special criminal law to reform environmental criminal law; or imitate France and incorporate environmental crimes into the environmental code under the background of China’s compilation of environmental laws. However, this subversive reform obviously cannot be realized in the short term. Referring to the legislative model of German environmental criminal law may be a more realistic choice, which means separating the legislation of environmental crime into an independent chapter in China’s penal code.

Finally, the issue of restorative justice for environmental crimes in China deserves more attention. In the judicial practice of the Environmental Pollution Crime, if the defendant can actively restore the damaged ecological environment, he or she may receive a certain degree of lenience in punishment. In addition to the traditional payment of ecological restoration fees, the defendants can also choose a variety of non-monetary restoration measures, such as replanting the vegetation, reclaiming the land, breeding and releasing, and purifying the waters [64]. Restorative justice for environmental crimes has ecological restoration effects that cannot be achieved by traditional penal methods (such as fixed-term imprisonment). Of course, the measure also faces the question of “spending money on punishment”. In this regard, China’s Supreme People’s Procuratorate pointed out that the goal of punishing environmental crimes is not simply for the purposes of criminal punishment but to also to repair social relations, restore the ecological environment, and show a benign development trend. The deputy procurator general of China’s Supreme People’s Procuratorate believes that allowing the parties to take the initiative to restore the ecology is not only an investigation of his legal responsibility, but also a positive guide to society [65]. In view of this, China’s environmental crimes can give more consideration to the adoption of restorative justice models in future punishments.

## 5. Conclusions

Although there is still a huge gap in the development of countries in the world, since the modern environmental movement in the middle of the 20th century, the importance of environmental protection has been deeply rooted in the hearts of the people all over the world, and the significance of global environmental governance has been increasingly recognized. In the past 50 years, global environmental governance has gone through three stages. Major developing countries, such as China and India will play an increasingly important role in the future global environmental governance. As one of the five permanent members of the United Nations and the largest greenhouse gas emitter in the world, the effectiveness of China’s environmental protection will greatly affect the effectiveness of global environmental governance and the living environment of all mankind. [66]

At the substantive level, China’s environmental protection work began in the 1970s. However, since then, China has not achieved the environmental protection goals set by itself over a relatively long historical period, which is closely related to the lack of a strong guarantee mechanism for environmental governance in China at that time. After entering the 21st century, China’s rulers and legislators are obviously aware of this problem. [67] This study attempts to illustrate that, from the perspective of global environmental governance, whether in response to international environmental protection pressures or to meet the needs of the Chinese people for a better life, the status of environmental protection as China’s national development strategy has been increasingly solid. In the past, China’s environmental crisis has continued to worsen, and the main reason for the lack of influence of China’s environmental law is that China’s punishment for environmental crimes is not as strong as it should be. In recent years, after China has strengthened its crackdown on environmental crimes, especially the punishment of Environmental Pollution Crime, China’s environmental conditions and peoples’ environmental protection awareness have been significantly improved, and the deterrent effect of China’s environmental laws has also increased significantly. In the process of China’s continuous promotion of ecological civilization construction, the legislation and judicial practice of China’s environmental crimes should be continuously optimized so as to ensure the implementation of China’s environmental protection strategy.

The environmental protection history of the world’s major industrialized countries is very similar. As a civil law country, China should vigorously learn from the environmental crime punishment experience of environmental protection pioneer countries represented by Germany and Japan and at the same time implement localization. By doing so, it could not only make positive contributions to China’s environmental protection cause, and at the same time contribute China’s wisdom, strategies and plans to global environmental governance.

## Data Availability

Not applicable.
